# 

**Published:** 1956-10

**Authors:** 


### Contents

| | | Page |
|---|---|---|
| VISITING PROFESSORS | *Editorial* | 119 |
| VISIT TO IRAQ AND TURKEY | *Professor G. Gordon Lennon* | 120 |
| STUDENT APPRENTICESHIP TO GENERAL PRACTITIONERS | *Mr. W. M. Capper* | 124 |
| MODERN TRENDS IN PUBLIC HEALTH | *Dr. R. C. Wofinden* | 126 |
| DOMENIQUE JEAN LARREY, 1766–1842 | *Dr. A. E. S. Roberts* | 134 |
| "EUNUCHS DO NOT TAKE THE GOUT" | *Dr. G. D. Kersley* | 136 |
| ISLET CELL TUMOUR OF THE PANCREAS | *Clinical Pathological Conference* | 138 |
| OBITUARY | | 142 |
| NOTES AND NEWS | | 142 |
| NEWS FROM SOCIETIES | | 143 |
| APPOINTMENTS | | 148 |

